# The One Percent Stroke: A Case Report

**DOI:** 10.7759/cureus.89062

**Published:** 2025-07-30

**Authors:** Sibi Ganesan, Sakthi Shanmugasundaram P, Swathy Moorthy, Emmanuel Bhaskar

**Affiliations:** 1 General Medicine, Sri Ramachandra Institute of Higher Education and Research, Chennai, IND; 2 Internal Medicine, Sri Ramachandra Institute of Higher Education and Research, Chennai, IND

**Keywords:** cardiac myxoma, central retinal vein occlusion, cerebrovascular accident, embolic stroke, intracardiac mass, left atrial tumor, myxoma excision, neurological deficits, rare stroke etiology

## Abstract

Cardiac myxoma (CM) is the most prevalent form of benign primary tumor of the heart, but it is a rare cause of stroke, accounting for less than 1% of strokes in the general population. CM has a heterogeneous clinical presentation that can frequently resemble other cardiovascular or neurological disorders. This diminutive differential will often impede timely diagnosis. We describe a 42-year-old woman who presented with right-sided hemiparesis and aphasia of sudden onset. Neuroimaging findings were significant for multiple acute non-hemorrhagic infarcts on the left side of the brain, possibly suggesting an embolic source. Echocardiography findings were compatible with a left atrial myxoma. The patient subsequently developed central retinal vein occlusion (CRVO) in the left eye. The patient was provided with medical and supportive care to include anticoagulation, and she underwent successful resection/excision of the left atrial myxoma, which subsequently led to a partial neurological recovery. This case highlights important considerations for cardiac sources of embolic strokes in young patients who do not have more traditional vascular risk factors. Rapid use of echocardiography to identify rare but treatable causes like CM should be considered. Successful surgical management of CM represents an effective approach. Failure to complete successful surgical management raises the risk of further side effects from other occult sources of emboli. CM should be a consideration in the differential diagnosis of embolic strokes in young adults, even if it is a rare etiology. Timely multimodal imaging, combined with proactive clinical efforts, should result in better patient outcomes.

## Introduction

Cardiac myxoma (CM), the most common primary benign tumor of the heart, is a gradually advancing neoplasm of endocardial origin [1]. The diagnosis of cardiac myxoma in young stroke patients is often difficult because it can manifest in different ways, including non-specific cardiac symptoms, embolic events (approximately 50% have cerebral embolism), and constitutional symptoms [2]. Our case describes a left atrial myxoma, presenting with an acute shower of emboli causing neurological deficits.

## Case presentation

A 42-year-old female patient, a homemaker with no previously known comorbidities, with no prior health check-ups or treatment, with no previous family cardiac history, with a BMI of 26 kg/m^2 ^(overweight), presented to the outpatient department with complaints of sudden-onset weakness of the right upper and lower limbs since the previous evening. The patient was apparently normal until 3 pm prior to the day of admission, when she attended a funeral during which she noted numbness over the left upper and lower limbs; she sat down for a little while and returned home, ignoring the symptom as it was not severe enough. At home, she had gone to use the restroom at 4 pm. The patient was found on the restroom floor at 5 pm by the patient attender, conscious, not responding to calls, and unable to get up. She also noted to be unable to use her right upper and lower limbs. She was rushed to a local hospital, and on the way, the patient had multiple episodes of vomiting. She was drowsy, conscious, and not responding to commands. A non-contrast CT brain done in the outside hospital showed multiple non-hemorrhagic infarcts involving the left cerebrum. The patient was started on oral antiplatelet drugs and referred to our hospital for further management the next day.

On examination, the patient was drowsy but arousable. Vitals were stable. Her neurological examination revealed ptosis of the left eye, with anisocoria. The left pupil was 3 mm, not reacting to light, whereas the right pupil was 2 mm, sluggishly reacting to light. Fundoscopy revealed left-sided papilledema. She was aphasic, and motor system examination revealed 0/5 power in her right upper and lower limbs. However, she was able to move her left upper and lower limbs against gravity to oral command with a power of 4-/5. Her other systemic examination was unremarkable.

**Figure 1:**
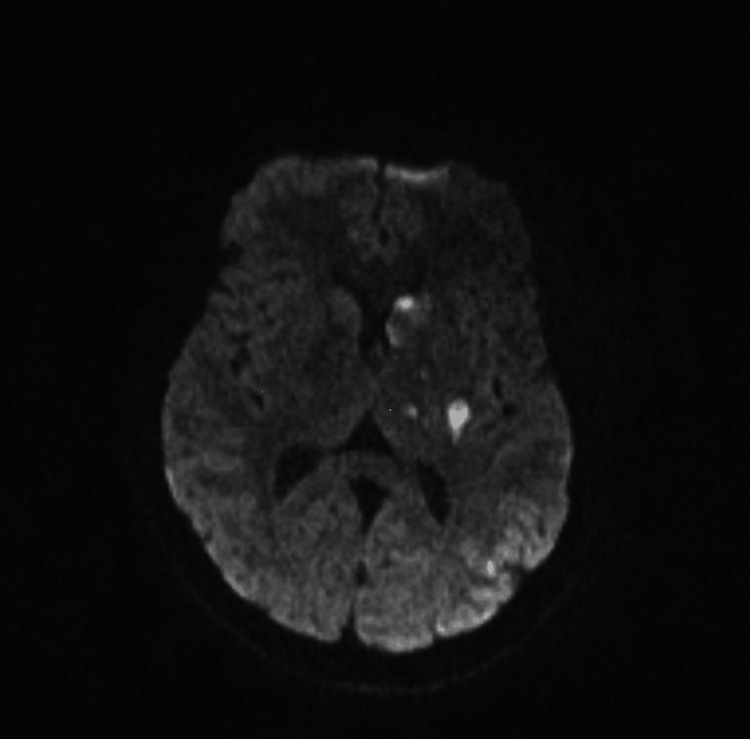
MRI brain showing multiple acute non-hemorrhagic infarcts involving the left cerebral hemisphere with features suggestive of raised intracranial tension in the left frontal region

**Figure 2:**
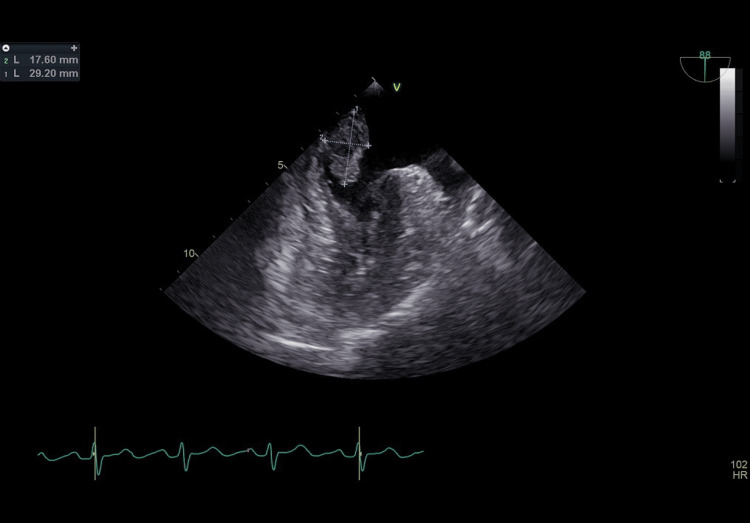
Transesophageal echo showing left atrial myxoma attached to the IAS of size 25*14mm (pedunculated) IAS: interatrial septum.

The patient was admitted to the intensive care unit. Suspecting raised intracranial pressure (ICP), 100 mL of 3% NaCl was given, and the patient was taken for an urgent MRI brain, stroke protocol, which showed multiple acute non-hemorrhagic infarcts involving the left cerebral hemisphere with features suggestive of raised intracranial tension in the left frontal region (Figure 1). The patient was started on anticoagulation (Enoxaparin) and antiplatelets, with a provisional diagnosis of embolic cerebrovascular accident (CVA) with right hemiparesis and motor aphasia. Her basic blood workup was within normal limits. Two-dimensional transthoracic echocardiography (ECHO) done showed a left atrial (LA) myxoma attached to the interatrial septum (IAS) of size 25*14mm, pedunculated, which was confirmed on a transesophageal echocardiogram (Figure 2). Twenty-four-hour Holter done showed normal study.

The following day, the patient had an improvement in power of the right upper and lower limbs to about 2/5 with improving verbal output. On further evaluation, the patient was noted to have no perception of light in the left eye. A repeat fundus screening was sought, and the patient was noted to have features suggestive of central retinal vein occlusion (CRVO). Poor visual prognosis of the left eye was explained to the patient and her family. Left atrial myxoma excision was done, and the patient was discharged home. At discharge, the patient is conscious and oriented. Power in the upper limb on the right side improved to 4/5.

## Discussion

Myxomas are one of the most frequent primary cardiac tumors of endocardial origin. The prevalence of atrial myxoma is less than five cases per 10,000 cases. Histologically, these tumors consist of dispersed cells inside a mucopolysaccharide stroma. The cells are derived from a multipotent mesenchyme with the potential for neuronal and endothelial development. These cells remain as embryonic remnants throughout cardiac septation and differentiate into endothelial cells, smooth muscle cells, angioblasts, fibroblasts, chondrocytes, and myoblasts. The occurrence of myxomas in the atrial septum is thus comprehensible [3]. Myxomas secrete vascular endothelial growth factor, likely aiding in angiogenesis and the initial phases of tumor development [4].

Cardiac myxomas are characterized by the combination of intracardiac obstruction, embolic signs, and systemic symptoms. They may remain totally asymptomatic, present with typical symptoms, or lead to life-threatening emergencies due to systemic embolization or even sudden cardiac death, depending on the location, size, morphology, and histopathology of the myxoma when there is systemic embolization [5]. A French study of 112 cases of cardiac myxomas summarized that intracardiac obstruction in the form of mitral valve obstruction was the most common manifestation (67%), followed by embolization (29%) and constitutional symptoms (34%) [1].

However, cardiac myxoma presenting as a stroke is relatively rare with cardiac myxomas being responsible for only 0.5% of stroke, with females at the age of 50 years at greatest risk [6]. This was true in our patient, a female patient in the fifth decade of life presenting as cerebrovascular accident. Although myxomas occur more often in women, men exhibited a higher likelihood of showing signs of embolization. In myxomas, the occurrence of embolization relates to smaller sizes (≤4.5 cm) and more pliable tumors [7].

In a retrospective cohort study for benign atrial neoplasms, conducted at the University Hospital of Tübingen between 2005 and 2017, in which 52 cases of atrial myxoma were diagnosed, only 13 presented with cerebrovascular events (CVE) were noted. Of whom, eight (62%) presented with ischemic stroke. Another three (23%) presented with transient ischemic attacks, and two patients (15%) with retinal ischemia. Only one patient (0.7%) was diagnosed with ischemic stroke with secondary, partial hemorrhagic transformation [8]. Our patient too had evidence of ischemic stroke, aphasia, and central retinal vein occlusion. In a similar case published from Canada in 2003, a 48-year-old male complained of acute neurological deficits and subsequently underwent a workup which led to a diagnosis of a left atrial myxoma. The tumor was surgically excised, and he made an impressive recovery neurologically after the procedure [9].

Recent literature highlights that although cardiac myxomas are histologically benign, they act functionally malignant because of their potential for embolization and systemic inflammation. A recent narrative review published in 2022 suggested that >50% of patients with atrial myxomas might first present with embolic complications, mostly ischemic strokes, regardless if they had cardiac symptoms [10]. Our case is similar to many other myxoma cases that the patient presented primarily with neurologic deficits and a retinal vein occlusion, from which his intermittent cardiovascular complaint had preceded. Additionally, the same review proposed that constitutional symptoms, such as low-grade fevers and fatigue, may result from elevated levels of interleukin-6 produced by the tumor, which could complicate early diagnosis as well [10]. In our patient, the absence of systemic symptoms emphasizes that early echocardiographic assessment should take place in younger patients presenting with unexplained strokes. The review also highlights that surgical excision is the first line and is associated with an excellent prognosis when done in a timely manner [10]. Following this suggestion, our patient had successful surgical excision of the left atrial mass shortly after the diagnosis and made significant improvements in both motor strength and verbal output. This case therefore corroborates the present consensus that early diagnosis and ultimately surgical definitive treatment are essential to prevent recurrent embolic events, systemic complications, and sudden cardiac death. Our patient's successful postoperative outcome suggests that cardiac etiology should be considered in stroke of unknown etiology when presented with a stroke of an isolated nature like our patient, particularly in younger patients with no traditional risk factors.

These case studies are quite rare but highlight the importance of being vigilant for the possibility of cardiac myxoma in any patient, especially younger patients who present with stroke and have no traditional vascular risk factors (hypertension, diabetes, atrial fibrillation) [1,4,6,8,9]. Echocardiography, and particularly transesophageal echocardiography (TEE), remains the best test to assess for cardiac mass lesions and should be considered as part of the path for evaluation in cryptogenic strokes especially for patients aged less than 55. Timely recognition and surgical intervention not only improve neurological outcomes but importantly decrease risk of embolic recurrences, sudden cardiac death, and systemic complications of intracardiac obstruction. Cardiac surgery however carries a low morbidity of approximately 5%, and later recurrences are virtually eliminated [11].

## Conclusions

Atrial myxoma, while uncommon, should be recognized as an important and potentially treatable cause of ischemic stroke, especially in younger patients without traditional vascular risk factors. Our case adds to the literature, which continues to highlight the diverse and sometimes elusive presentation of cardiac myxomas. This case indicates that timely recognition and treatment can lead to fewer complications or sequelae. Echocardiography, including transesophageal echocardiography, is still the gold standard for diagnosis and should be performed early in the workup of patients who present with a cryptogenic cause for a stroke. This case reinforces the need for increased clinical vigilance for potential cardiac sources of embolism when investigating stroke patients without a clear etiology. Early cardiac investigations and surgical management can reduce the risk of recurrent embolic events and may help improve functional recovery and overall prognosis.
